# A Multidimensional Evaluation of Large Language Model Responses to the 2024 ESC Hypertension Guidelines: A Comparative Study

**DOI:** 10.3390/jcdd13100481

**Published:** 2026-09-24

**Authors:** Ferit Böyük, Aysun Karahan Gün, İsmail Polat Canbolat, Emre Özmen, Harun Akarsu, Halime Tanrıverdi, Bilal Cuğlan, Kemal Türker Ulutaş

**Affiliations:** 1 Department of Cardiology, Kanuni Sultan Süleyman Training and Research Hospital, Health Science University, İstanbul 34303, Türkiye; aysun.karahan4@saglik.gov.tr (A.K.G.); ismail.canbolat@saglik.gov.tr (İ.P.C.); emre.ozmen1@saglik.gov.tr (E.Ö.); harun.akarsu@saglik.gov.tr (H.A.); bilal.cuglan@sbu.edu.tr (B.C.); 2 Department of Cardiology, Hatay Reyhanlı State Hospital, Hatay 31500, Türkiye; halime.tanriverdi@saglik.gov.tr; 3 Department of Biochemistry, Hatay Reyhanlı State Hospital, Hatay 31500, Türkiye; kemalturker.ulutas@saglik.gov.tr

**Keywords:** large language models, hypertension, ESC guideline, medical education, clinical decision support

## Abstract

Large language models (LLMs), including ChatGPT, Gemini, and DeepSeek, are increasingly used in medicine; however, their performance across multiple clinically relevant domains remains incompletely understood. This cross-sectional comparative study evaluated 225 responses generated by three LLMs to 75 clinical questions selected from the 2024 European Society of Cardiology (ESC) Hypertension Guidelines. Responses were independently assessed by two board-certified cardiologists across five predefined domains: accuracy, clinical relevance, completeness, absence of bias and misinformation, and consistency. No statistically significant differences were observed among the three models in accuracy, clinical relevance, completeness, or absence of bias and misinformation (all *p* > 0.05). A significant difference was identified in response consistency (*p* = 0.032), with post hoc analysis demonstrating a significant difference between ChatGPT and Gemini. Overall appropriateness scores did not differ significantly among the three LLMs (*p* = 0.227). These findings suggest that, although overall performance was comparable, response consistency represents an additional dimension that should be considered when evaluating LLMs for guideline-based clinical applications. Future studies incorporating broader clinical scenarios and updated LLM versions are warranted to further define their role in clinical decision support.

## 1. Introduction

Hypertension, a chronic and asymptomatic disease, is a significant cause of mortality and morbidity worldwide. Most of the time, organ damage has already begun when hypertension is diagnosed. Early diagnosis of hypertension is essential, and it depends heavily on patient education and treatment. Therefore, rapid and complete access to accurate information by healthcare professionals and patients may positively affect clinical outcomes in the long term [1,2,3,4].

In recent years, large language models (LLMs) such as ChatGPT, Gemini, and Deepseek have become increasingly popular in healthcare due to their fast and easy access to medical information. Such models are used not only in accessing information but also in medical education and patient communication. Although language models provide advantages regarding fast access to information, issues such as the accuracy and completeness of the information provided raise serious concerns. The ease of public access to language models also brings additional problems, such as the spread of misinformation. In recent years, the success of language models in medical applications and medical education has been the subject of research. Still, the multidimensional examination of real clinical scenarios based on guidelines among these language models is limited [5,6,7,8].

Recent investigations have highlighted that LLMs can achieve high accuracy on standardized medical licensing exams and retrieve guidelines for cardiovascular care. However, these studies predominantly evaluated raw accuracy alone [9]. Our study addresses this gap by executing a multidimensional evaluation—incorporating consistency and completeness—specifically focused on the newly published 2024 ESC Hypertension Guidelines.

In this study, the responses of three major LLMs mentioned above to 75 clinical questions based on the 2024 ESC hypertension guideline were examined in a multidimensional and systematic manner, and their performances were compared according to the answers given by LLMs. The answers given by LLMs were compared in terms of accuracy, clinical relevance, completeness, bias, misinformation, and consistency. The performance of the language models according to the answers given by these LLMs to a global health problem (e.g., hypertension) was examined multidimensionally, and performance characteristics of the AI models were revealed. This study contributes to the ongoing discussions regarding integrating LLMs into medical education and clinical decision-making.

## 2. Methods

### 2.1. Study Design and Objective

This cross-sectional and comparative study aims to systematically and multidimensionally investigate the performance of three open LLMs: ChatGPT, Gemini, and DeepSeek. Within this overarching goal, the responses of three language models to 75 questions based on the 2024 ESC hypertension guideline were examined.

### 2.2. Question Selection and AI Response Generation

A total of 75 clinical questions were selected from the major topics covered in the 2024 ESC Guidelines for the management of elevated blood pressure and hypertension. The questions were chosen to provide broad representation of the guideline, including blood pressure measurement, diagnosis, cardiovascular risk assessment, lifestyle interventions, pharmacological treatment, follow-up, and special clinical situations. The number of questions was determined a priori to ensure comprehensive coverage of the guideline rather than based on a formal statistical sample size calculation. Therefore, this study was designed as an exploratory, guideline-based comparative evaluation of large language model responses.

The 75 clinical questions were generated by the investigators to sample the core components of the 2024 ESC Hypertension Guidelines. To ensure broad coverage, the questions were distributed across major clinical themes: Blood Pressure Measurement and Diagnosis (*n* = 19), Cardiovascular Risk Stratification (*n* = 15), Lifestyle Interventions (*n* = 9), Pharmacological Initiation and Targets (*n* = 14), and Resistant, Secondary, and Special Clinical Situations (*n* = 18).

These three specific LLMs were selected because they represent prominent publicly accessible LLM platforms developed by separate artificial intelligence research laboratories (OpenAI, Google, and DeepSeek). Their inclusion allowed comparison of user-facing LLM systems from different developers. The models evaluated during the study period (March 2025) were ChatGPT (GPT-4o), Gemini (Gemini 1.5 Pro), and DeepSeek (DeepSeek-V3). All prompts were submitted through their respective web-based user interfaces, and no user-adjustable generation parameters or system configurations were modified, thereby assessing the default out-of-the-box response behaviors of each platform.

All questions were entered as written text without modification into ChatGPT, Gemini, and DeepSeek during the same study period (March 2025). To minimize potential carry-over effects from previous interactions, each question was submitted in a new chat session for each LLM. The same question was presented sequentially to all three models, and only the initial response generated by each model was included in the analysis. No follow-up prompts, clarifications, or response regeneration were performed. All responses were anonymized before evaluation, retrieved from the system, and recorded in JSON format for subsequent analysis.

### 2.3. Evaluation Criteria

The answers given to each question were evaluated in 5 different categories based on the HT guidelines. The accuracy of the answers given by the models to similar questions was tested. The study evaluated not only the accuracy of the responses but also their clinical relevance, completeness, absence of bias and misinformation, and consistency. The five-domain evaluation framework was not adopted from a single formally validated instrument. Rather, these domains were informed by evaluation dimensions reported in previous peer-reviewed literature assessing artificial intelligence responses and clinical guideline compliance.

1. Accuracy: Accuracy was evaluated based on conceptual and clinical recommendation alignment with the 2024 ESC Guidelines rather than exact textual or keyword matching. Responses that employed alternative wording or clinical synonyms but conveyed the same clinical recommendation, target, or classification were categorized as appropriate (1). Conversely, responses providing outdated blood pressure targets or unapproved therapeutic pathways were categorized as inappropriate (0).
2. Clinical Relevance: Clinical relevance assessed whether the LLM provided directly actionable clinical information that addressed the question. To receive an appropriate score (1), the model’s response had to offer practical utility for the clinical scenario while remaining free of non-feasible diagnostic steps or irrelevant or non-specific information.
3. Completeness: Completeness was measured by evaluating whether the LLM response incorporated the core diagnostic or therapeutic recommendations essential to the specific clinical scenario. A response was categorized as inappropriate (0) if it omitted key diagnostic steps or essential therapeutic instructions required to satisfy the clinical pathway outlined in the 2024 ESC Guidelines.
4. Absence of Bias and Misinformation: This domain assessed whether the response was free of unsupported preferential or commercially biased recommendations, fabricated citations, and factually incorrect pharmacological or clinical information. Responses containing fabricated or unsupported claims, incorrect trial or drug information, or materially preferential framing not supported by the guideline were categorized as inappropriate (0).
5. Consistency was assessed by evaluating the stability and coherence of each model’s recommendations across questions addressing related clinical concepts. Responses were categorized as inappropriate (0) when clinically meaningful recommendations conflicted across related queries without a scenario-based explanation.

A binary scoring system was used for each domain (appropriate = 1; inappropriate = 0) to provide a uniform domain-level classification across responses and minimize multi-tier scoring complexity. The five domain scores were summed to generate a total score ranging from 0 to 5 for each response. Supplementary Table S2 provides a descriptive operationalization of the qualitative characteristics underlying appropriate and inappropriate classifications.

The responses were independently evaluated by two board-certified cardiologists. Overall appropriateness scores were reported as median with minimum and maximum values (range). Table 1 below shows an illustrative evaluation example. The complete scoring dataset across all 75 questions is provided in Supplementary Table S1.

### 2.4. Statistical Analysis

Categorical variables (appropriate vs. inappropriate) were summarized as frequencies and percentages. Overall appropriateness scores were reported as median with minimum and maximum values (range). Inter-rater reliability between the two cardiologists before consensus reconciliation was assessed using Cohen’s kappa (κ).

Comparisons among the three LLMs were performed using Cochran’s Q test for paired binary outcomes. When Cochran’s Q test was statistically significant, post hoc pairwise comparisons were performed using McNemar’s test. Differences in overall appropriateness scores were assessed using the Friedman test for related samples. All statistical tests were two-sided, and a *p*-value < 0.05 was considered statistically significant. Statistical analyses were performed using IBM SPSS Statistics for Windows, Version 25.0 (IBM Corp., Armonk, NY, USA).

## 3. Results

### 3.1. Domain-Specific Appropriateness

A total of 75 clinical questions selected from the 2024 European Society of Cardiology (ESC) Hypertension Guidelines were submitted to three large language models (LLMs), ChatGPT, DeepSeek, and Gemini, yielding 225 AI-generated responses. Each response was independently evaluated across five predefined domains: accuracy, clinical relevance, completeness, absence of bias and misinformation, and consistency. Before consensus reconciliation, inter-rater agreement between the two cardiologists was strong (Cohen’s κ = 0.82).

As presented in Table 2, no statistically significant differences were observed among the three LLMs in the domains of accuracy (*p* = 0.297), clinical relevance (*p* = 0.773), completeness (*p* = 0.826), or absence of bias and misinformation (*p* = 0.704) according to Cochran’s Q test.

A statistically significant difference was observed in the consistency domain (*p* = 0.032). Post hoc pairwise comparisons using McNemar’s test demonstrated a significant difference between ChatGPT and Gemini (*p* = 0.014), whereas no significant differences were observed between ChatGPT and DeepSeek (*p* = 0.164) or between DeepSeek and Gemini (*p* = 0.442).

The cross-model agreement patterns across the five evaluation domains are presented in Table 3.

### 3.2. Total Appropriateness Scores

Each response was assigned an overall appropriateness score ranging from 0 to 5 based on the sum of the five evaluation domain scores. As shown in Table 4, the median overall appropriateness scores were 2 (range, 0–5) for ChatGPT, 4 (range, 1–4) for DeepSeek, and 3 (range, 1–5) for Gemini. The overall appropriateness rates for each model are presented in Figure 1.

## 4. Discussion

This study provides a comparative evaluation of three widely used large language models (ChatGPT, Gemini, and DeepSeek) using 75 clinical questions selected from the 2024 ESC Hypertension Guidelines. Responses were assessed across five predefined domains, including accuracy, clinical relevance, completeness, absence of bias and misinformation, and consistency. Although no statistically significant differences were observed in overall appropriateness scores or in most evaluation domains, a significant difference was identified in response consistency, with ChatGPT showing greater variability than Gemini. These findings suggest that, in addition to accuracy, response consistency represents an important characteristic when evaluating the performance of LLMs in guideline-based clinical questions [10,11,12]. Given the exploratory nature of this study, these pairwise findings should be interpreted with caution and confirmed in larger studies.

The differences in response consistency observed among the evaluated LLMs warrant further investigation. Although these models are based on similar transformer architectures, variations in response consistency may be related to differences in training data, model optimization, inference strategies, or other proprietary design characteristics. Because these underlying factors are not publicly available, the reasons for the observed differences cannot be determined from the present study and should therefore be interpreted with caution [13,14]. The observed differences in response consistency should not be interpreted as evidence of overall superiority of one LLM over another. Instead, they may reflect differences in model architecture, training data, optimization strategies, or other proprietary design characteristics. As these technical aspects are not fully disclosed by the developers, the underlying reasons for the observed variability remain uncertain and warrant further investigation [15]. These findings highlight the importance of evaluating not only the accuracy but also the consistency of LLM-generated responses before their integration into clinical practice. Accurate responses alone may not be sufficient if similar clinical questions yield inconsistent recommendations. Although the present study did not assess clinical outcomes, variability in response consistency could have implications for user confidence and decision support in clinical settings, particularly when LLMs are used without expert supervision.

Although our analysis identified statistical differences in consistency scores, these findings reflect differences in the stability of responses across related queries rather than direct impacts on clinical outcomes. Future investigations are required to establish whether variations in LLM consistency translate into differences in clinical decision support quality or patient safety [16]. Furthermore, the implications of LLM output consistency may vary based on clinical expertise. Experienced cardiologists may be better positioned to recognize omissions or factual errors in inconsistent AI outputs. Conversely, trainee physicians or medical students may be more vulnerable to inconsistent AI recommendations. While our study did not empirically measure user behaviors or clinical outcomes, the theoretical risk of trainees adopting unverified AI outputs underscores the need for cautious implementation in educational environments [17].

Our findings are consistent with previous studies reporting that LLMs perform well on medical examinations and guideline-based questions. However, our study extends the existing literature by demonstrating that response consistency may differ among LLMs even when overall appropriateness is comparable. These findings suggest that consistency should be considered as an additional performance metric in future evaluations of LLMs for clinical applications [12,18,19]. Previous studies have also highlighted the limitations of LLMs in clinical applications. For example, Gilson et al. and Cascella et al. reported that the fluent and convincing language generated by ChatGPT may mask deficiencies in the underlying medical content, emphasizing the need for cautious interpretation of LLM-generated responses in clinically important decision-making contexts [15,16,17,18,19].

Previous studies have primarily focused on the accuracy of LLM-generated responses to medical questions. Although accuracy remains a fundamental requirement for the clinical use of LLMs, other performance domains, including consistency, clinical relevance, completeness, and the absence of bias and misinformation, should also be considered. Accordingly, our study adopted a multidimensional evaluation framework that assessed these complementary aspects in addition to accuracy. This broader approach may contribute to a more comprehensive assessment of LLM performance in guideline-based clinical applications and may inform future evaluation frameworks [20].

## 5. Clinical Implications

The findings of this study suggest that the evaluation of LLMs for clinical use should extend beyond response accuracy alone. Additional performance domains, particularly response consistency, may provide complementary information when assessing the reliability of LLM-generated recommendations. Although overall appropriateness was comparable among the evaluated models, the observed differences in consistency indicate that responses to similar clinical questions may vary across LLMs.

Accordingly, LLM-generated responses should be interpreted as supportive information rather than as a substitute for clinical judgment or evidence-based guidelines. Further studies across diverse clinical scenarios, medical specialties, and prospective settings are needed to determine the clinical significance of response variability and to evaluate the role of LLMs in clinical decision support.

## 6. Limitations

This study has several limitations. First, responses were evaluated by two board-certified cardiologists, and although independent assessments followed by consensus were performed, some degree of subjective interpretation cannot be completely excluded. Second, the study was based exclusively on questions selected from the 2024 ESC Hypertension Guidelines; therefore, the findings may not be generalizable to other medical specialties or clinical guidelines. Third, the study was designed as an exploratory evaluation using a predefined set of 75 guideline-based questions rather than a formally calculated sample size. Fourth, responses were evaluated using a binary scoring system, which may not fully capture differences in the quality or nuance of LLM-generated answers. Furthermore, while opening a new chat session for each question minimizes intra-conversational context carry-over, subtle backend caching or cross-session optimization by proprietary LLM providers cannot be entirely controlled.

Finally, LLMs are continuously updated, and the present findings reflect model performance only during the study period (March 2025). Therefore, future studies including a broader range of clinical scenarios, updated model versions, and more comprehensive evaluation methods are warranted.

## 7. Conclusions

This study provides a multidimensional comparison of three publicly accessible large language models using guideline-based clinical questions derived from the 2024 ESC Hypertension Guidelines. Although the three LLMs demonstrated comparable overall appropriateness, differences in response consistency were observed. These findings suggest that the evaluation of LLMs for medical applications should extend beyond accuracy alone and should also consider consistency, clinical relevance, completeness, and the absence of bias and misinformation. Future studies incorporating broader clinical scenarios, updated LLM versions, and more comprehensive evaluation frameworks are needed to better define the role of LLMs in clinical decision support.

## Figures and Tables

**Figure 1:**
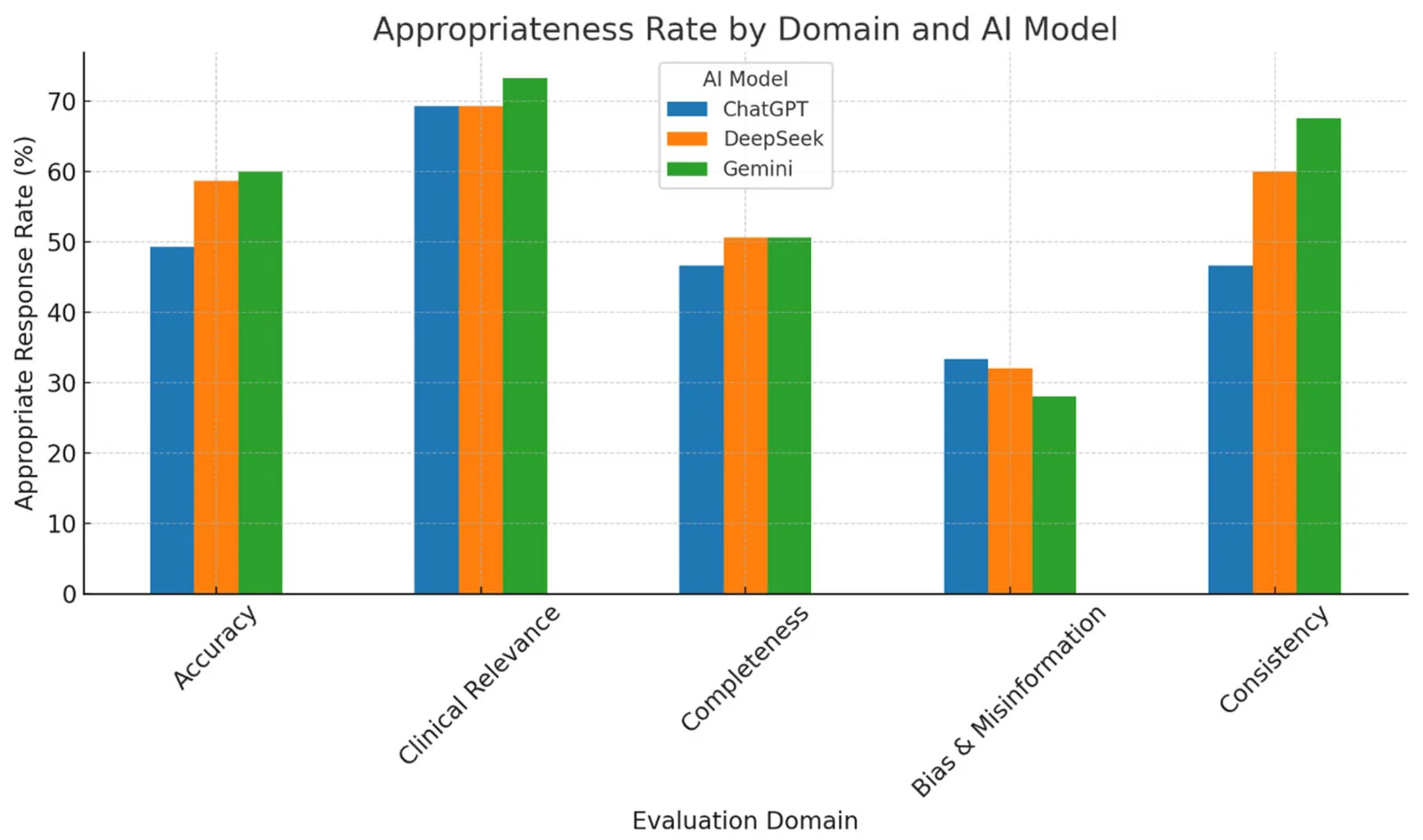
Domain-Specific Appropriateness Rates Across the Three Large Language Models.

**Table 1:** Example of the Response Evaluation Framework.

Question Example	AI Model	Accuracy	Clinical Relevance	Completeness	Bias and Misinformation	Consistency	Total Score (0–5)
How can out-of-office BP measurements help in detecting white-coat hypertension and masked hypertension?	ChatGPT	✓	✓	✓	✗	✓	4
	DeepSeek	✓	✓	✓	✗	✓	4
	Gemini	✓	✓	✓	✗	✓	4
What is recommended if out-of-office BP measurements are not feasible for confirming a diagnosis?	ChatGPT	✓	✓	✓	✗	✓	4
	DeepSeek	✓	✓	✓	✗	✓	4
	Gemini	✓	✓	✓	✗	✓	4

✓ = Appropriate; ✗ = Inappropriate.

**Table 2:** Appropriateness of AI-Generated Responses Across Five Evaluation Domains.

Evaluation Domain	AI Model	Inappropriate n (%)	Appropriate n (%)	*p*-Value	Pairwise Comparisons (*p*)
Accuracy	ChatGPT	38 (50.7%)	37 (49.3%)	0.297	–
	DeepSeek	31 (41.3%)	44 (58.7%)		
	Gemini	30 (40.0%)	45 (60.0%)		
Clinical Relevance	ChatGPT	23 (30.7%)	52 (69.3%)	0.773	–
	DeepSeek	23 (30.7%)	52 (69.3%)		
	Gemini	20 (26.7%)	55 (73.3%)		
Completeness	ChatGPT	40 (53.3%)	35 (46.7%)	0.826	–
	DeepSeek	37 (49.3%)	38 (50.7%)		
	Gemini	37 (49.3%)	38 (50.7%)		
Absence of Bias and Misinformation	ChatGPT	50 (66.7%)	25 (33.3%)	0.704	–
	DeepSeek	51 (68.0%)	24 (32.0%)		
	Gemini	54 (72.0%)	21 (28.0%)		
Consistency	ChatGPT	40 (53.3%)	35 (46.7%)	0.032	CGPT–DS: 0.164
	DeepSeek	30 (40.0%)	45 (60.0%)		CGPT–G: 0.014
	Gemini	25 (33.3%)	50 (66.7%)		DS–G: 0.442

**Table 3:** Cross-model Agreement Matrix Across Evaluation Domains.

Evaluation Domain	CGPT:Inap//DS:App	CGPT:App/DS:Inap	CGPT:Inap/DS:Inap	CGPT:App/DS:App	CGPT:Inap/G:App	CGPT:App/G:Inap	CGPT:Inap/G:Inap	CGPT:App/G:App	DS:Inap/G:App	DS:App/G:Inap	DS:Inap/G:Inap	DS:App/G:App
Accuracy	23 (30.7%)	16 (21.3%)	15 (20.0%)	21 (28.0%)	18 (24.0%)	10 (13.3%)	20 (26.7%)	27 (36.0%)	14 (18.7%)	13 (17.3%)	17 (22.7%)	31 (41.3%)
Clinical Relevance	13 (17.3%)	13 (17.3%)	10 (13.3%)	39 (52.0%)	15 (20.0%)	12 (16.0%)	8 (10.7%)	40 (53.3%)	10 (13.3%)	7 (9.3%)	13 (17.3%)	45 (60.0%)
Completeness	20 (26.7%)	17 (22.7%)	20 (26.7%)	18 (24.0%)	15 (20.0%)	12 (16.0%)	25 (33.3%)	38 (30.7%)	15 (20.0%)	15 (20.0%)	22 (29.3%)	23 (30.7%)
Bias and Misinformation	13 (17.3%)	14 (18.7%)	37 (49.3%)	11 (14.7%)	12 (16.0%)	16 (21.3%)	38 (50.7%)	9 (12.0%)	8 (10.7%)	11 (14.7%)	43 (57.3%)	13 (17.3%)
Consistency	26 (34.7%)	16 (21.3%)	14 (18.7%)	19 (25.3%)	24 (32.0%)	9 (12.0%)	16 (21.3%)	26 (34.7%)	16 (21.3%)	11 (14.7%)	14 (18.7%)	34 (45.3%)

Abbreviations: CGPT = ChatGPT, DS = DeepSeek, G = Gemini; Inap = Inappropriate, App = Appropriate.

**Table 4:** Total Appropriateness Scores by Model.

AI Model	Median (Min–Max)
ChatGPT (n = 75)	2 (0–5)
DeepSeek (n = 75)	4 (1–4)
Gemini (n = 75)	3 (1–5)

Statistical Comparison: Friedman test, *p* = 0.227.

## Data Availability

The datasets generated and analyzed during the current study are available from the corresponding author upon reasonable request. The verbatim outputs from all three AI models (ChatGPT, Gemini, and DeepSeek) that were analyzed in this study are provided in the Supplementary Materials.

## Supplementary Materials

The following supporting information can be downloaded at: https://www.mdpi.com/article/10.3390/jcdd13100481/s1.

## References

[1] Martín-Fernández J., Alonso-Safont T., Polentinos-Castro E., Esteban-Vasallo M.D., Ariza-Cardiel G., González-Anglada M.I., Sánchez-Perruca L., Rodríguez-Martínez G., Rotaeche-del-Campo R., Bilbao-González A. (2023). Impact of hypertension diagnosis on morbidity and mortality: A retrospective cohort study in primary care. BMC Prim. Care.

[2] McEvoy J.W., McCarthy C.P., Bruno R.M., Brouwers S., Canavan M.D., Ceconi C., Christodorescu R.M., Daskalopoulou S.S., Ferro C.J., Gerdts E. (2024). 2024 ESC Guidelines for the management of elevated blood pressure and hypertension. Eur. Heart J..

[3] Ukoha-Kalu B.O., Isah A., Biambo A.A., Samaila A., Abubakar M.M., Kalu U.A., Soyiri I.N. (2023). Effectiveness of educational interventions on hypertensive patients’ self-management behaviours: An umbrella review protocol. BMJ Open.

[4] Ren Y., Wang Z., Wang Q. (2024). The trend of hypertension-related chronic kidney disease from 1990 to 2019 and its predictions over 25 years: An analysis of the Global Burden of Disease Study 2019. Int. Urol. Nephrol..

[5] Abd-Alrazaq A., AlSaad R., Alhuwail D., Ahmed A., Healy P.M., Latifi S., Aziz S., Damseh R., Alabed Alrazak S., Sheikh J. (2023). Large language models in medical education: Opportunities, challenges, and future directions. JMIR Med. Educ..

[6] Skryd A., Lawrence K. (2024). ChatGPT as a tool for medical education and clinical decision-making on the wards: Case study. JMIR Form. Res..

[7] Nazi Z.A., Peng W. (2024). Large language models in healthcare and medical domain: A review. Informatics.

[8] Kung T.H., Cheatham M., Medenilla A., Sillos C., Leon L.D., Elepaño C., Madriaga M., Aggabao R., Diaz-Candido G., Maningo J. (2023). Performance of ChatGPT on USMLE: Potential for AI-assisted medical education using large language models. PLoS Digit. Health.

[9] Cascella M., Montomoli J., Bellini V., Bignami E. (2023). Evaluating the feasibility of ChatGPT in healthcare: An analysis of multiple clinical and research scenarios. J. Med. Syst..

[10] Nerella S., Bandyopadhyay S., Zhang J., Contreras M., Siegel S., Bumin A., Silva B., Sena J., Shickel B., Bihorac A. (2023). Transformers in healthcare: A survey. Artif. Intell. Med..

[11] Yeo Y.H., Samaan J.S., Ng W.H., Ting P.-S., Trivedi H., Vipani A., Ayoub W., Yang J.D., Liran O., Spiegel B. (2023). Assessing the performance of ChatGPT in answering questions regarding cirrhosis and hepatocellular carcinoma. Clin. Mol. Hepatol..

[12] Wang L., Chen X., Deng X., Wen H., You M.K., Liu W.Z., Li Q., Li J. (2024). Prompt engineering in consistency and reliability with the evidence-based guideline for LLMs. npj Digit. Med..

[13] Jiao W., Wang W., Huang J., Wang X., Shi S., Tu Z. (2023). Is ChatGPT a good translator? Yes with GPT-4 as the engine. arXiv.

[14] Brown T., Mann B., Ryder N., Subbiah M., Kaplan J.D., Dhariwal P., Neelakantan A., Shyam P., Sastry G., Askell A. (2020). Language models are few-shot learners. NeurIPS.

[15] Nori H., King N., McKinney S.M., Carignan D., Horvitz E. (2023). Capabilities of gpt-4 on medical challenge problems. arXiv.

[16] Hager P., Jungmann F., Holland R., Bhagat K., Hubrecht I., Knauer M., Vielhauer J., Makowski M., Braren R., Kaissis G. (2024). Evaluation and mitigation of the limitations of large language models in clinical decision-making. Nat. Med..

[17] Lucas H.C., Upperman J.S., Robinson J.R. (2024). A systematic review of large language models and their implications in medical education. Med. Educ..

[18] Gilson A., Safranek C.W., Huang T., Socrates V., Chi L., Taylor R.A., Chartash D. (2023). How does ChatGPT perform on the United States Medical Licensing Examination (USMLE)? The implications of large language models for medical education and knowledge assessment. JMIR Med. Educ..

[19] Lee P., Bubeck S., Petro J. (2023). Benefits, limits, and risks of GPT-4 as an AI chatbot for medicine. N. Engl. J. Med..

[20] Ghassemi M., Oakden-Rayner L., Beam A.L. (2021). The false hope of current approaches to explainable artificial intelligence in health care. Lancet Digit. Health.

