# Supplementary material for: A Multidimensional Evaluation of Large Language Model Responses to the 2024 ESC Hypertension Guidelines: A Comparative Study
Source: J Cardiovasc Dev Dis. 2026 Sep 24;13(10):481. doi: 10.3390/jcdd13100481 (PMC13705208; doi:10.3390/jcdd13100481)
Supplement: Supplementary file 1 [file jcdd-13-00481-s001.zip › jcdd-4463084_Supplementary_Table_S2.pdf]

Supplementary Table S2. Operational characteristics used for the five-domain evaluation framework.

Evaluation Domain	Appropriate (1) Characteristics	Inappropriate (0) Characteristics	Illustrative Description
**Accuracy**	Conceptually concordant with the relevant 2024 ESC recommendation(s), without clinically meaningful factual errors.	Inclusion of outdated thresholds, contradictory data, or incorrect targets.	Aligning with target blood pressure ranges vs. providing obsolete cutoff metrics.
**Clinical Relevance**	Provides directly actionable information addressing the clinical question.	Recommends non-feasible pathways or generic medical descriptions lacking utility.	Providing specific clinically applicable information vs. providing irrelevant or non-specific information.
**Completeness**	Includes the clinically essential diagnostic or therapeutic elements needed to adequately address the clinical scenario.	Omits key diagnostic steps or essential therapeutic instructions.	Detailing the complete procedural steps for confirmation vs. omitting standard diagnostic path requirements.
**Absence of Bias and Misinformation**	Contains no fabricated or unsupported medical claims and no materially unsupported preferential framing that distorts the guideline-based recommendation.	Exhibits systematic preference for specific commercial products over guideline alternatives, introduces non-existent trials, or outputs hallucinated numerical metrics.	Maintaining objective, guideline-based information vs. generating fictional clinical references or unverified drug preferences.
**Consistency**	No clinically meaningful contradiction across responses addressing comparable clinical concepts.	Displays contradictory stances or severe variance on identical core concepts.	Withstanding stable recommendation patterns across related queries vs. generating conflicting directives.
