# Supplementary material for: A Multidimensional Evaluation of Large Language Model Responses to the 2024 ESC Hypertension Guidelines: A Comparative Study
Source: J Cardiovasc Dev Dis. 2026 Sep 24;13(10):481. doi: 10.3390/jcdd13100481 (PMC13705208; doi:10.3390/jcdd13100481)
Supplement: Supplementary file 1 [file jcdd-13-00481-s001.zip › jcdd-4463084_Supplementary_Table_S1.pdf]

Supplementary Table S1. Full Evaluation of AI-Generated Responses Across 75 Hypertension Questions

Question	AI Model	Accuracy	Clinical Relevance	Completeness	Bias & Misinformation	Consistency	Total Score
1. How can out-of-office BP measurements help in detecting white-coat hypertension and masked hypertension?	ChatGPT<br>DeepSeek<br>Gemini	✓<br>✓<br>✓	✓<br>✓<br>✓	✓<br>✓<br>✓	✗<br>✗<br>✗	✓<br>✓<br>✓	4<br>4<br>4
2. What is recommended if out-of-office BP measurements are not feasible for confirming a diagnosis?	ChatGPT<br>DeepSeek<br>Gemini	✓<br>✓<br>✓	✓<br>✓<br>✓	✓<br>✓<br>✓	✗<br>✗<br>✗	✓<br>✓<br>✓	4<br>4<br>4
3. How should a diagnosis of hypertension be confirmed if out-of-office measurements are not possible?	ChatGPT<br>DeepSeek<br>Gemini	✓<br>✓<br>✓	✓<br>✓<br>✓	✓<br>✓<br>✓	✗<br>✗<br>✗	✓<br>✓<br>✓	4<br>4<br>4
4. Why have most automated oscillometric monitors not been validated for BP measurement in AF?	ChatGPT<br>DeepSeek<br>Gemini	✓<br>✓<br>✓	✓<br>✓<br>✓	✓<br>✓<br>✓	✗<br>✗<br>✗	✓<br>✓<br>✓	4<br>4<br>4
5. What BP measurement method should be used in patients with atrial fibrillation if automated oscillometric monitors are not validated?	ChatGPT<br>DeepSeek<br>Gemini	✓<br>✓<br>✓	✓<br>✓<br>✓	✓<br>✓<br>✓	✗<br>✗<br>✗	✓<br>✓<br>✓	4<br>4<br>4
6. Why is a manual auscultatory method recommended for BP measurement in AF patients?	ChatGPT<br>DeepSeek<br>Gemini	✓<br>✓<br>✓	✓<br>✓<br>✓	✓<br>✓<br>✓	✗<br>✗<br>✗	✓<br>✓<br>✓	4<br>4<br>4
7. When should an assessment for orthostatic hypotension be considered?	ChatGPT<br>DeepSeek<br>Gemini	✗<br>✗<br>✗	✓<br>✓<br>✗	✗<br>✗<br>✗	✗<br>✗<br>✓	✗<br>✓<br>✗	1<br>2<br>1
8. What is the criteria for diagnosing orthostatic hypotension?	ChatGPT<br>DeepSeek<br>Gemini	✗<br>✓<br>✗	✓<br>✓<br>✓	✗<br>✗<br>✗	✗<br>✗<br>✗	✗<br>✓<br>✗	1<br>3<br>1
9. How should the orthostatic hypotension assessment be performed?	ChatGPT<br>DeepSeek<br>Gemini	✓<br>✓<br>✗	✓<br>✓<br>✓	✓<br>✓<br>✗	✗<br>✗<br>✗	✓<br>✓<br>✓	4<br>4<br>2
10. What approach is recommended for the treatment of elevated BP?	ChatGPT<br>DeepSeek<br>Gemini	✗<br>✗<br>✗	✗<br>✓<br>✗	✗<br>✗<br>✗	✓<br>✗<br>✓	✗<br>✗<br>✗	1<br>1<br>1
11. Which individuals are considered at increased risk for CVD events?	ChatGPT<br>DeepSeek<br>Gemini	✓<br>✗<br>✗	✓<br>✓<br>✓	✓<br>✗<br>✗	✗<br>✗<br>✗	✓<br>✗<br>✗	4<br>1<br>1
12. Why are individuals with certain conditions considered at increased risk for CVD events?	ChatGPT<br>DeepSeek<br>Gemini	✗<br>✓<br>✗	✓<br>✓<br>✗	✗<br>✓<br>✗	✗<br>✗<br>✓	✗<br>✓<br>✗	1<br>4<br>1
13. What is the recommended CVD risk threshold for individuals with elevated BP to be considered at increased risk for CVD?	ChatGPT<br>DeepSeek<br>Gemini	✗<br>✓<br>✓	✓<br>✓<br>✓	✗<br>✓<br>✗	✗<br>✗<br>✗	✗<br>✓<br>✓	1<br>4<br>3

Question	AI Model	Accuracy	Clinical Relevance	Completeness	Bias & Misinformation	Consistency	Total Score
14. Does the recommended CVD risk threshold for risk-based management of elevated BP vary by age?	ChatGPT<br>DeepSeek<br>Gemini	✗<br>✗<br>✗	✗<br>✗<br>✗	✗<br>✗<br>✗	✓<br>✓<br>✓	✗<br>✗<br>✗	1<br>1<br>1
15. What should be the basis for the risk-based management of elevated BP?	ChatGPT<br>DeepSeek<br>Gemini	✓<br>✓<br>✗	✓<br>✓<br>✗	✗<br>✓<br>✗	✗<br>✗<br>✓	✗<br>✓<br>✗	2<br>4<br>1
16. What tool should be used to estimate CVD risk in type 2 diabetes mellitus patients with elevated BP?	ChatGPT<br>DeepSeek<br>Gemini	✓<br>✓<br>✓	✓<br>✓<br>✓	✓<br>✓<br>✓	✗<br>✗<br>✓	✓<br>✓<br>✓	4<br>4<br>5
17. For which group of patients is SCORE2-Diabetes particularly recommended?	ChatGPT<br>DeepSeek<br>Gemini	✓<br>✓<br>✓	✓<br>✓<br>✓	✓<br>✓<br>✗	✗<br>✗<br>✗	✓<br>✓<br>✓	4<br>4<br>3
18. Why is SCORE2-Diabetes particularly recommended for patients under 60 years of age?	ChatGPT<br>DeepSeek<br>Gemini	✓<br>✓<br>✓	✓<br>✓<br>✓	✓<br>✓<br>✓	✗<br>✗<br>✗	✓<br>✓<br>✓	4<br>4<br>4
19. What are some pregnancy complications that should be considered as sex-specific risk modifiers for individuals with elevated BP?	ChatGPT<br>DeepSeek<br>Gemini	✓<br>✓<br>✓	✓<br>✓<br>✓	✗<br>✓<br>✗	✗<br>✗<br>✗	✗<br>✓<br>✗	2<br>4<br>2
20. How do pregnancy complications affect the management of individuals with elevated BP and borderline increased 10-year CVD risk?	ChatGPT<br>DeepSeek<br>Gemini	✓<br>✗<br>✓	✓<br>✓<br>✓	✗<br>✗<br>✓	✗<br>✗<br>✗	✗<br>✓<br>✓	2<br>2<br>4
21. What is the recommended level of evidence for considering pregnancy complications as risk modifiers in individuals with elevated BP and borderline CVD risk?	ChatGPT<br>DeepSeek<br>Gemini	✗<br>✗<br>✗	✗<br>✗<br>✗	✗<br>✗<br>✗	✗<br>✓<br>✓	✗<br>✗<br>✓	0<br>1<br>2
22. How often should adults under 40 years of age be screened for elevated BP and hypertension?	ChatGPT<br>DeepSeek<br>Gemini	✗<br>✓<br>✗	✗<br>✓<br>✗	✗<br>✓<br>✗	✓<br>✗<br>✓	✗<br>✓<br>✗	1<br>4<br>1
23. How often should adults aged 40 years or older be screened for elevated BP and hypertension?	ChatGPT<br>DeepSeek<br>Gemini	✓<br>✓<br>✓	✓<br>✓<br>✓	✓<br>✓<br>✓	✗<br>✗<br>✗	✓<br>✓<br>✓	4<br>4<br>4
24. What is the purpose of the recommended screening intervals for BP and hypertension?	ChatGPT<br>DeepSeek<br>Gemini	✓<br>✗<br>✓	✓<br>✗<br>✓	✓<br>✗<br>✓	✓<br>✓<br>✗	✓<br>✗<br>✓	5<br>1<br>4
25. What should be considered for individuals with elevated BP who do not currently meet risk thresholds for BP-lowering treatment?	ChatGPT<br>DeepSeek<br>Gemini	✗<br>✓<br>✓	✗<br>✓<br>✓	✗<br>✓<br>✓	✗<br>✗<br>✗	✗<br>✓<br>✓	0<br>4<br>4
26. Why is a repeat BP measurement and risk assessment recommended within 1 year for individuals with elevated BP?	ChatGPT<br>DeepSeek<br>Gemini	✓<br>✗<br>✓	✓<br>✓<br>✓	✓<br>✗<br>✓	✗<br>✗<br>✗	✓<br>✗<br>✓	4<br>1<br>4
27. What should be done for individuals with increased CVD risk and office BP of 120–139/70–89 mmHg?	ChatGPT<br>DeepSeek<br>Gemini	✗<br>✓<br>✓	✗<br>✓<br>✓	✗<br>✓<br>✓	✓<br>✗<br>✗	✗<br>✓<br>✓	1<br>4<br>4

Question	AI Model	Accuracy	Clinical Relevance	Completeness	Bias & Misinformation	Consistency	Total Score
28. What should be done if out-of-office BP measurement methods are not logistically feasible?	ChatGPT<br>DeepSeek<br>Gemini	✓<br>✓<br>✓	✓<br>✓<br>✓	✓<br>✗<br>✓	✗<br>✗<br>✗	✓<br>✓<br>✓	4<br>3<br>4
29. Why is out-of-office BP measurement recommended for individuals with increased CVD risk?	ChatGPT<br>DeepSeek<br>Gemini	✓<br>✗<br>✓	✓<br>✓<br>✓	✓<br>✗<br>✓	✗<br>✗<br>✗	✓<br>✗<br>✓	4<br>1<br>4
30. What should be considered in the clinical work-up of patients with apparent resistant hypertension?	ChatGPT<br>DeepSeek<br>Gemini	✗<br>✓<br>✓	✗<br>✓<br>✓	✗<br>✓<br>✗	✓<br>✗<br>✗	✗<br>✓<br>✓	1<br>4<br>3
31. Why is objective evaluation of adherence recommended for patients with apparent resistant hypertension?	ChatGPT<br>DeepSeek<br>Gemini	✓<br>✗<br>✓	✓<br>✓<br>✓	✓<br>✗<br>✓	✗<br>✗<br>✗	✓<br>✗<br>✓	4<br>1<br>4
32. When should objective evaluation of adherence be considered for patients with resistant hypertension?	ChatGPT<br>DeepSeek<br>Gemini	✗<br>✓<br>✓	✗<br>✓<br>✓	✗<br>✓<br>✓	✓<br>✗<br>✗	✗<br>✓<br>✓	1<br>4<br>4
33. How often should serum creatinine, eGFR, and urine ACR be measured in individuals with moderate-to-severe CKD?	ChatGPT<br>DeepSeek<br>Gemini	✓<br>✗<br>✗	✓<br>✗<br>✗	✓<br>✗<br>✗	✗<br>✓<br>✓	✓<br>✗<br>✗	4<br>1<br>1
34. When may coronary artery calcium scoring be considered in patients with elevated BP or hypertension?	ChatGPT<br>DeepSeek<br>Gemini	✓<br>✗<br>✗	✓<br>✗<br>✗	✓<br>✗<br>✗	✗<br>✓<br>✓	✓<br>✗<br>✗	4<br>1<br>1
35. Who should be considered for screening for primary aldosteronism?	ChatGPT<br>DeepSeek<br>Gemini	✗<br>✓<br>✓	✗<br>✓<br>✓	✗<br>✗<br>✓	✓<br>✗<br>✗	✗<br>✗<br>✓	1<br>2<br>4
36. Why should opportunistic screening with office BP measurements be considered during late childhood and adolescence?	ChatGPT<br>DeepSeek<br>Gemini	✓<br>✓<br>✓	✓<br>✓<br>✓	✗<br>✓<br>✓	✗<br>✗<br>✗	✗<br>✓<br>✓	2<br>4<br>4
37. Why should potassium intake be increased in patients with hypertension and high daily sodium intake?	ChatGPT<br>DeepSeek<br>Gemini	✓<br>✓<br>✓	✓<br>✓<br>✓	✓<br>✓<br>✓	✗<br>✗<br>✗	✓<br>✓<br>✓	4<br>4<br>4
38. Why is it important to take medications at the most convenient time of day for the patient?	ChatGPT<br>DeepSeek<br>Gemini	✓<br>✗<br>✗	✓<br>✗<br>✗	✓<br>✗<br>✗	✗<br>✓<br>✓	✓<br>✗<br>✗	4<br>1<br>1
39. What level of CVD risk qualifies for BP lowering through lifestyle measures?	ChatGPT<br>DeepSeek<br>Gemini	✓<br>✗<br>✓	✓<br>✗<br>✓	✓<br>✗<br>✗	✗<br>✓<br>✗	✓<br>✗<br>✓	4<br>1<br>3
40. When is pharmacological treatment recommended for adults with elevated BP and high CVD risk?	ChatGPT<br>DeepSeek<br>Gemini	✗<br>✓<br>✓	✗<br>✓<br>✓	✗<br>✓<br>✓	✓<br>✗<br>✗	✗<br>✓<br>✓	1<br>4<br>4
41. What should be initiated in hypertensive patients with confirmed BP ≥140/90 mmHg, regardless of CVD risk?	ChatGPT<br>DeepSeek<br>Gemini	✓<br>✓<br>✗	✓<br>✓<br>✗	✓<br>✓<br>✗	✗<br>✗<br>✓	✓<br>✓<br>✗	4<br>4<br>1

Question	AI Model	Accuracy	Clinical Relevance	Completeness	Bias & Misinformation	Consistency	Total Score
42. Under what condition should BP-lowering drug treatment be maintained beyond the age of 85?	ChatGPT	✗	✓	✓	✗	✓	3
	DeepSeek	✗	✗	✗	✓	✗	1
	Gemini	✗	✗	✗	✓	✗	1
43. Under what conditions should BP-lowering treatment be considered for individuals with BP ≥140/90 mmHg?	ChatGPT	✗	✗	✗	✓	✗	1
	DeepSeek	✗	✗	✗	✓	✗	1
	Gemini	✗	✗	✗	✓	✗	1
44. What is recommended if BP-lowering treatment is poorly tolerated and achieving a target systolic of 120–129 mmHg is not possible?	ChatGPT	✗	✗	✗	✓	✗	1
	DeepSeek	✗	✗	✗	✓	✗	1
	Gemini	✓	✓	✓	✗	✓	4
45. When is comprehensive screening for the main causes of secondary hypertension recommended?	ChatGPT	✗	✗	✗	✓	✗	1
	DeepSeek	✗	✗	✗	✓	✗	1
	Gemini	✓	✓	✓	✗	✓	4
46. Why should HBPM and ABPM be considered in pregnancy?	ChatGPT	✓	✓	✓	✗	✓	4
	DeepSeek	✗	✗	✗	✓	✗	1
	Gemini	✗	✗	✗	✓	✗	1
47. How should elevated BP and hypertension be treated in older patients aged <85 years who are not frail?	ChatGPT	✓	✓	✓	✗	✓	4
	DeepSeek	✗	✗	✗	✓	✗	1
	Gemini	✓	✓	✓	✗	✓	4
48. What is the preferred sequence of medications for BP-lowering in frail or elderly patients?	ChatGPT	✗	✗	✗	✓	✗	1
	DeepSeek	✓	✓	✓	✗	✓	4
	Gemini	✓	✓	✓	✗	✓	4
49. How should orthostatic hypotension be tested before adjusting BP-lowering treatment?	ChatGPT	✗	✗	✗	✓	✗	1
	DeepSeek	✗	✗	✗	✓	✗	1
	Gemini	✓	✓	✓	✗	✓	4
50. Why are SGLT2 inhibitors recommended for hypertensive patients with CKD and eGFR >20 mL/min/1.73 m²?	ChatGPT	✓	✓	✓	✗	✓	4
	DeepSeek	✗	✓	✗	✓	✗	2
	Gemini	✓	✓	✓	✗	✓	4
51. When is BP-lowering drug treatment recommended for people with pre-diabetes or obesity?	ChatGPT	✗	✓	✗	✗	✓	2
	DeepSeek	✓	✓	✓	✗	✓	4
	Gemini	✓	✓	✗	✗	✓	3
52. What condition is specifically mentioned for renal artery angioplasty without stenting in hypertensive patients?	ChatGPT	✓	✓	✓	✗	✓	4
	DeepSeek	✗	✗	✗	✓	✗	1
	Gemini	✓	✓	✓	✗	✓	4
53. What is the recommended approach for BP reduction in patients with intracerebral haemorrhage and systolic BP ≥220 mmHg?	ChatGPT	✗	✗	✗	✓	✗	1
	DeepSeek	✗	✗	✗	✓	✗	1
	Gemini	✗	✗	✗	✓	✗	1
54. When should SCORE2 be used for assessing 10-year CVD risk?	ChatGPT	✗	✓	✗	✓	✗	2
	DeepSeek	✓	✓	✗	✗	✓	3
	Gemini	✓	✓	✗	✗	✓	3
55. When should SCORE2-OP be used to assess 10-year CVD risk?	ChatGPT	✗	✓	✗	✗	✗	1
	DeepSeek	✗	✗	✗	✓	✗	1
	Gemini	✓	✓	✗	✗	✓	3

Question	AI Model	Accuracy	Clinical Relevance	Completeness	Bias & Misinformation	Consistency	Total Score
56. What should be done when screening office BP is ≥160/100 mmHg?	ChatGPT	✗	✗	✗	✓	✗	1
	DeepSeek	✓	✓	✓	✗	✓	4
	Gemini	✓	✓	✓	✗	✓	4
57. Why is echocardiography recommended in patients with hypertension?	ChatGPT	✗	✓	✗	✗	✗	1
	DeepSeek	✓	✓	✓	✗	✓	4
	Gemini	✓	✓	✓	✗	✓	4
58. What BMI range is recommended to reduce BP and CVD risk?	ChatGPT	✗	✓	✗	✓	✗	2
	DeepSeek	✓	✓	✓	✗	✓	4
	Gemini	✗	✗	✗	✓	✗	1
59. What waist circumference values should be targeted to reduce BP and CVD risk?	ChatGPT	✓	✓	✓	✗	✓	4
	DeepSeek	✓	✓	✓	✗	✓	4
	Gemini	✓	✓	✓	✗	✓	4
60. What is the recommended alcohol consumption limit for men and women to reduce health risks?	ChatGPT	✗	✓	✗	✓	✗	2
	DeepSeek	✗	✗	✗	✓	✗	1
	Gemini	✗	✗	✗	✓	✗	1
61. How much alcohol is typically contained in one standard drink?	ChatGPT	✗	✓	✓	✗	✗	2
	DeepSeek	✓	✓	✓	✗	✓	4
	Gemini	✗	✓	✓	✗	✓	3
62. Which BP-lowering drugs are recommended as first-line treatments to reduce BP and CVD events?	ChatGPT	✓	✓	✓	✗	✓	4
	DeepSeek	✓	✓	✓	✗	✓	4
	Gemini	✓	✓	✓	✗	✓	4
63. What is the recommended target for systolic BP to reduce CVD risk in most adults?	ChatGPT	✗	✗	✗	✓	✗	1
	DeepSeek	✓	✓	✗	✗	✓	3
	Gemini	✗	✓	✗	✗	✓	2
64. What should be considered if systolic BP is at or below target but diastolic BP is ≥80 mmHg?	ChatGPT	✗	✓	✗	✗	✗	1
	DeepSeek	✓	✓	✗	✗	✓	3
	Gemini	✓	✓	✓	✗	✓	4
65. What are the criteria for considering personalized, more lenient BP targets in patients with hypertension?	ChatGPT	✗	✗	✗	✓	✗	1
	DeepSeek	✗	✗	✗	✓	✗	1
	Gemini	✗	✓	✗	✗	✓	2
66. In which patients is renal denervation not recommended for treating hypertension?	ChatGPT	✗	✓	✗	✓	✗	2
	DeepSeek	✓	✓	✓	✗	✓	4
	Gemini	✗	✓	✗	✗	✓	2
67. When should drug treatment be started in women with gestational hypertension?	ChatGPT	✗	✗	✗	✓	✗	1
	DeepSeek	✗	✗	✗	✓	✗	1
	Gemini	✗	✗	✗	✓	✗	1
68. What is the goal for diastolic BP in women with chronic and gestational hypertension?	ChatGPT	✓	✓	✓	✗	✓	4
	DeepSeek	✗	✗	✗	✓	✗	1
	Gemini	✗	✗	✗	✓	✗	1
69. When should pharmacological treatment for BP be initiated in adults with elevated BP and diabetes?	ChatGPT	✗	✗	✗	✓	✗	1
	DeepSeek	✗	✗	✗	✓	✗	1
	Gemini	✗	✗	✗	✓	✗	1

Question	AI Model	Accuracy	Clinical Relevance	Completeness	Bias & Misinformation	Consistency	Total Score
70. What is the recommended target systolic BP for individuals with diabetes who are receiving BP-lowering drugs?	ChatGPT	✗	✗	✗	✓	✗	1
	DeepSeek	✓	✓	✓	✗	✓	4
	Gemini	✗	✓	✗	✗	✗	1
71. What is recommended for patients with moderate-to-severe CKD and confirmed BP ≥130/80 mmHg?	ChatGPT	✓	✓	✓	✗	✓	4
	DeepSeek	✗	✗	✗	✓	✗	1
	Gemini	✓	✓	✓	✗	✓	4
72. What is the recommended target systolic BP for adults with moderate-to-severe CKD and eGFR >30 mL/min/1.73 m² who are receiving BP-lowering drugs?	ChatGPT	✗	✓	✗	✗	✗	1
	DeepSeek	✓	✓	✓	✗	✓	4
	Gemini	✗	✓	✗	✗	✗	1
73. What level of evidence supports the use of SGLT2 inhibitors in hypertensive patients with symptomatic HFpEF?	ChatGPT	✗	✗	✗	✗	✗	0
	DeepSeek	✓	✓	✓	✗	✓	4
	Gemini	✓	✓	✓	✗	✓	4
74. What is the recommended target systolic BP for patients with intracerebral haemorrhage within 6 hours of symptom onset?	ChatGPT	✓	✓	✓	✗	✓	4
	DeepSeek	✓	✓	✓	✗	✓	4
	Gemini	✗	✓	✗	✗	✗	1
75. What is the recommended drug treatment for severe hypertension in pregnancy?	ChatGPT	✓	✓	✓	✗	✓	4
	DeepSeek	✓	✓	✓	✗	✓	4
	Gemini	✓	✓	✓	✗	✓	4

Abbreviations: AI, artificial intelligence; BP, blood pressure; CVD, cardiovascular disease; CKD, chronic kidney disease; eGFR, estimated glomerular filtration rate; HFpEF, heart failure with preserved ejection fraction.

✓ indicates an appropriate classification (1); ✗ indicates an inappropriate classification (0).
